# Use of the prostate‐specific antigen test in the U.S. for men age 30 to 64 in 2011 to 2017 using a large commercial claims database: Implications for practice interventions

**DOI:** 10.1002/cnr2.1365

**Published:** 2021-05-02

**Authors:** Shahram Shahangian, Krishna P. Sharma, Lin Fan, David A. Siegel

**Affiliations:** ^1^ Division of Laboratory Systems CDC Atlanta Georgia USA; ^2^ Division of Cancer Prevention and Control CDC Atlanta Georgia USA

**Keywords:** mass screening, prostate‐specific antigen, prostatic neoplasm, public health practice

## Abstract

**Background:**

Given the public health relevance of PSA‐based screening, various professional organizations have issued recommendations on the use of the PSA test to screen for prostate cancer in different age groups.

**Aim:**

Using a large commercial claims database, we aimed to determine the most recent rates of PSA testing for privately insured men age 30 to 64 in the context of screening recommendations.

**Methods and Results:**

Data from employer plans were from MarketScan commercial claims database. Annual PSA testing rate was the proportion of men with ≥1 paid test(s) per 12 months of continuous enrollment. Men with diagnosis of any prostate‐related condition were excluded. Annual percent change (APC) in PSA test use was estimated using joinpoint regression analysis. In 2011 to 2017, annual testing rate encompassing 5.02 to 5.53 million men was approximately 1.4%, age 30 to 34; 3.4% to 4.1%, age 35 to 39; 11% to 13%, age 40 to 44; 18% to 21%, age 45 to 49; 31% to 33%, age 50 to 54; 35% to 37%, age 55 to 59; and 38% to 41%, age 60 to 64. APC for 2011 to 2017 was −0.5% (*P* = .11), age 30 to 34; −3.0% (*P* = .001), age 35‐39; −3.1% (*P* < .001), age 40 to 44; −2.4% (*P* = .001), age 45 to 49; −0.2% (*P* = .66), age 50 to 54; 0.0% (*P* = .997), age 55 to 59; and −3.3% (*P* = .054) from 2011 to 2013 and 1.2% (*P* = .045) from 2013 to 2017, age 60 to 64. PSA testing rate decreased from 2011 to 2017 for age groups between 35 and 49 by 13.4% to 16.9%.

**Conclusions:**

Based on these data, PSA testing rate has modestly decreased from 2011 to 2017. These results, however, should be considered in view of the limitation that MarketScan claims data may not be equated to actual PSA testing practices in the entire U.S. population age 30 to 64. Future research should be directed to understand why clinicians continue ordering PSA test for men younger than 50.

## 1 INTRODUCTION

The test for prostate‐specific antigen (PSA) has been used effectively in the monitoring of diagnosed prostate cancer; however, there have been differing guidelines in using this test to screen for prostate cancer, and the recommendations for screening practices have evolved over the years. In 2008, the U.S. Preventive Services Task Force (USPSTF) noted that there was insufficient evidence to screen men younger than 75.^1^ In 2012, the USPSTF recommended against PSA screening of all men for prostate cancer.^2^ Subsequent to the 2012 recommendation, the USPSTF released a draft recommendation in April 2017 noting that the potential benefits and harms of PSA‐based screening were closely balanced in men age 55 to 69 years, and that the decision whether to be screened should be an individual one.^3^ The USPSTF issued its most recent recommendation in May 2018,^4^ that was identical to the 2017 draft recommendation.^3^ The recommendations by the American Urological Association (AUA)^5^ and American College of Physicians (ACP)^6^ in 2013, and American Cancer Society (ACS) in 2016^7^ although consistent with current USPSTF recommendations,^4^ did not agree with the 2012 USPSTF recommendation against routine screening of all men.^2^ The AUA and USPSTF recommend offering to screen men age 55 to 69 for prostate cancer after a process of shared decision‐making.^4^, ^5^ The ACP recommends limiting routine screening to men age 50 to 69 with at least 10 years of life expectancy if they agree to be tested after discussions with their clinicians,^6^ while the ACS recommends PSA‐based prostate cancer screening of men age ≥ 50 with at least 10 years of life expectancy.^7^

In the context of PSA‐based screening recommendations for prostate cancer and given its public health relevance, the objective of this study was to determine the more recent rate and trend for annual PSA testing in a sample of privately insured U.S. men age 30 to 64 in 2011 through 2017 using a large insurance claims database. This study is unique in that it is, to our knowledge, the only report of PSA testing rates and trends through 2017 that also includes men in younger age groups between 30 and 49. The unique observation made in our study for the most recent PSA testing trends is that from 2017 to 2018, PSA testing increased for the first time in recent years. Information gained from this study may be used to inform needed practice interventions for younger men (age 30‐49) who should not be routinely screened for prostate cancer using the PSA test according to practice guidelines issued by various professional organization.

## 2 METHODS

We used claims data from large employer‐sponsored health plans obtained from IBM Watson Health's MarketScan databases from January 1, 2011 through April 30, 2019, encompassing 5.02 to 5.53 million men with continuous enrollment for each calendar year. These data represent a convenience sample of ~10% of the U.S. population, and they were captured using a web‐based tool provided by IBM called Treatment Pathways.^8^ For the PSA testing trend in 2011 to 2017, we used the January 1, 2011 to July 31, 2018 100%‐sample database. We further compared PSA testing rates in 2018 to those in 2017 by using the most recently released 100%‐sample database covering claims from January 1, 2012 to April 30, 2019 which became available to us after completion of our analyses for testing trends from 2011 to 2017. We excluded all claims with one or more diagnoses or non‐laboratory procedures relating to prostate‐related diseases or conditions including those diagnostic codes related to elevated PSA or history of prostate cancer (see Appendix). We did this to exclude encounter claims for PSA testing that was performed for differential diagnosis including benign prostatic hyperplasia, and for cancer monitoring or prognostic purposes. We included only claims with a Current Procedural Terminology (CPT, American Medical Association) Code 84152 for complexed PSA and Code 84153 for total PSA, and Healthcare Common Procedure Coding System (HCPCS, Centers for Medicare & Medicaid Services) code G0103 for total PSA testing. We did not use CPT code 84154 for free PSA because this test, unlike total or complexed PSA, is not initially used to screen for prostate cancer. If PSA test result is in the borderline range, usually considered 4.0 to 10.0 μg/L, free/total PSA ratio may be used to decide if a prostate biopsy is warranted.

### 2.1 Annual PSA testing rates

Annual rates for PSA test use in each calendar year were the proportion of included men with ≥1 PSA test(s) per 12 months continuous enrollment that were paid. Encounters in MarketScan databases reflect paid claims only, and unpaid claims are not captured.

### 2.2 Annual percent change and PSA testing trends

Annual percent change (APC) in PSA test use was estimated using joinpoint regression analysis, fitting trend data to identify the log‐linear model with the fewest number of inflection points.^9^ The APC was the log‐linear slope of each trend line, and the 2‐sided *P* values related to statistical significance (ie, *P* < .05) for each APC estimate being different from zero.

### 2.3 Stratification scheme

We stratified MarketScan claims into age 30 to 34, 35 to 39, 40 to 44, 45 to 49, 50 to 54, 55 to 59, and 60 to 64. To study regional variation in PSA testing rates, we further stratified claims data into the 4 U.S. Census regions (https://www.census.gov/prod/1/gen/95statab/preface.pdf). These regions are Northeast (NE): CT, MA, ME, NH, RI, NJ, NY, PA, and VT; Midwest (MW): IA, IL, IN, KS, MI, MN, MO, OH, NE, ND, SD, and WI; South (S): AL, AR, DC, DE, FL, GA, KY, LA, MD, MS, NC, OK, SC, VA, TN, TX, and WV; and West (W): AK, AZ, CA, CO, HI, ID, MT, NM, NV, OR, UT, WA, and WY.

### 2.4 Hemoglobin testing trends

To ascertain that the trends observed for PSA testing rate were not due to changes in clinical laboratory testing in general, we determined testing trends for hemoglobin (CPT Codes 83026, 83051, and 85018), a test not impacted by any changes in practice recommendations, as a measure of trend in laboratory testing.

## 3 RESULTS

### 3.1 Included populations

The number of men included in each year from 2011 through 2017 ranged from 4.55 to 5.14 million after excluding claim encounters with diagnostic or non‐laboratory procedure codes relating to prostate related diseases or conditions, including codes related to elevated PSA and history of prostate cancer. Consequently, 92.6% to 93.1% of men remained for analysis after this exclusion. Table 1 shows the age and regional distribution of men included in the analyses from 2011 to 2017. No regional assignment could be made for 0.2% to 0.3% of men due to missing data.

**TABLE 1:** Age and regional distribution of included men from 2011 through 2017^a^

	2011	2012	2013	2014	2015	2016	2017
N^b^	4.90	5.06	5.14	4.79	4.67	4.66	4.55
Age							
30–34	11.7%	13.1%	12.0%	12.2%	12.4%	12.7%	13.0%
35–39	13.6%	13.3%	13.4%	13.5%	13.5%	13.8%	14.0%
40–44	15.2%	15.4%	15.5%	15.5%	15.2%	14.8%	14.5%
45–49	16.5%	16.2%	16.0%	15.8%	15.7%	15.9%	16.0%
50–54	16.7%	16.7%	16.7%	16.8%	16.8%	16.5%	16.1%
55–59	15.2%	15.8%	15.6%	15.7%	15.8%	15.8%	15.7%
60–64	11.0%	10.7%	10.6%	10.5%	10.6%	10.5%	10.6%
Region							
NE	17.4%	17.2%	16.9%	18.1%	18.9%	19.0%	18.8%
MW	22.0%	21.5%	21.8%	22.4%	22.4%	22.2%	23.6%
S	39.2%	38.9%	39.0%	41.7%	40.4%	40.3%	40.3%
W	21.1%	21.8%	22.0%	17.5%	18.1%	18.3%	17.0%

^a^ NE, Northeast; MW, Midwest; S, South; W, West; ^b^N, number of enrollees in millions.

### 3.2 Annual PSA testing rates and percent changes

Annual rate of PSA testing ranged from approximately 1.4% in men age 30‐34 to 38%‐39% in men age 60‐64. Over the period from 2011 to 2017, PSA testing rate decreased by 2.7%, 16.5%, 16.9%, 13.4%, 2.1%, 1.3%, and 2.1%, respectively, for men age 30 to 34, 35 to 39, 40 to 44, 45 to 49, 50 to 54, 55 to 59, and 60 to 64. Table 2 shows the range of annual PSA testing rate and annual percent change (APC) for men in each of these seven groups.

**TABLE 2:** Annual PSA testing rate and annual percent change (APC)^a^

	Testing rate	APC	Years	P
Age				
30–34	1.36%–1.42%	−0.5%	2011–2017	.11
35–39	3.43%–4.14%	−3.0%	2011–2017	.001
40–44	11.0%–13.4%	−3.1%	2011–2017	<.001
45–49	18.1%–21.0%	−2.4%	2011–2017	.001
50–54	31.2%–33.0%	−0.2%	2011–2017	.66
55–59	34.7%–36.8%	.0%	2011‐2017	.997
60–64	37.6%–40.2%	−3.2%	2011‐2013	.054
60‐64	37.6%–39.3%	+1.2%	2013‐2017	.045

^a^ Testing rates are the ranges, that is, minimum to maximum rates, observed for annual rates from 2011 through 2017.

### 3.3 Annual PSA testing trends

**FIGURE 1:**
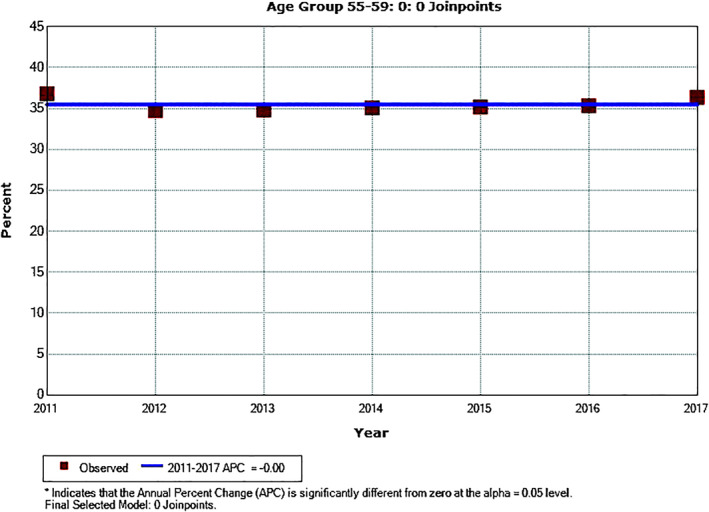
Annual PSA testing rates and trend in men age 55 to 59

**FIGURE 2:**
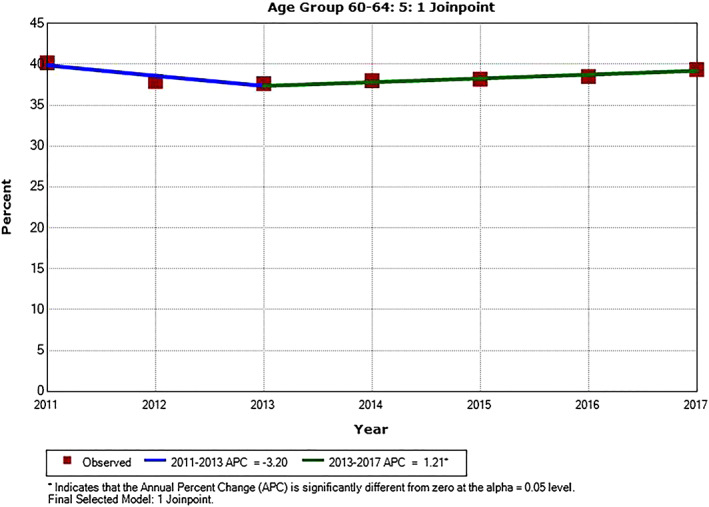
Annual PSA testing rates and trend in men age 60 to 64. ^*^APC is significantly different from zero (*P* < .05)

**FIGURE 3:**
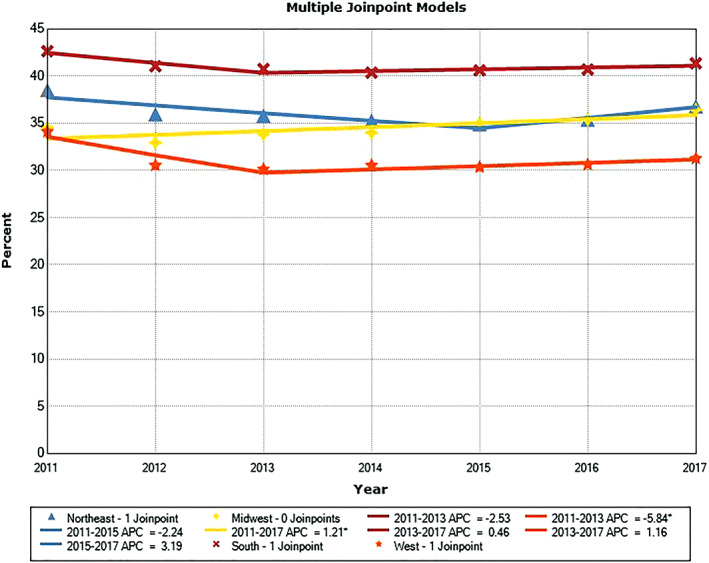
Annual PSA testing rates and trends in men age 55 to 64 stratified by Census region. ^*^APC is significantly different from zero (*P* < .05)

Except for PSA testing trend in men age 60 to 64, there was no inflection point in annual testing rate from 2011 to 2017. Annual testing showed significantly downward trends in men age 35 to 39 (*P* = .001), 40 to 44 (*P* < .001) and 45 to 49 (*P* = .001). There were no significant trends for PSA testing rate in men age 30 to 34 (*P* = .11), 50 to 54 (*P* = .66), and 55 to 59 (*P* = .997) from 2011 to 2017. For men age 60 to 64, PSA testing rate trended downward from 2011 to 2013 (*P* = 0.054) and upward from 2013 to 2017 (*P* = .045). The trends of annual PSA testing rates for men ages 55 to 59 and 60 to 64 are shown in Figures 1 and 2, respectively. Annual PSA testing rates and their trends in men age 55 to 64 are shown in Figure 3, after stratifying them by U.S. Census region. The highest testing rates are seen in the South, while the lowest rates are in the West. For the Southern and Western regions APC from 2011 to 2013 was −2.5% (*P* = .051) and −5.8% (*P* = .033), respectively; and from 2013 to 2017 it was +0.5% (*P* = .14) and +1.2% (*P* = .11), respectively. For the Northeastern region APC was −2.2% (*P* = .15) from 2011 to 2015; and it was +3.2% (*P* = .43) from 2015 to 2017. For the Midwestern region APC was +1.2% from 2011 to 2017 (*P* = .035).

### 3.4 PSA testing rates in 2018 compared to those in 2017

There were increases in PSA testing rates for all but two age groups from 2017 to 2018, ranging from 2.7% to 10.7%. Annual percent change was +9.5%, age 30 to 34; −2.4%, age 35 to 39; −0.5%, age 40 to 44; +2.7%, age 45 to 49; +5.1%, age 50 to 54; +7.8%, age 55 to 59; and +10.7%, age 60 to 64. For men age 40 to 49, PSA testing rate increased by 3.8% in the Northeast and 8.6% in the West but decreased by 1.2% in the Midwest and 0.1% in the South. For men age 55 to 64, PSA testing rate increased in all 4 regions. This increase was 11.4% in the Northeast, 10.5% in the Midwest, 6.4% in the South, 12.1% in the West.

### 3.5 Hemoglobin testing trends

None of the seven age groups or the eight age‐group/region combinations showed any inflection point from 2011 to 2017, and the slopes of the trend lines were not significantly different from zero (*P* values ranging from .070 to .96).

## 4 DISCUSSION

Several studies have assessed the annual rates for PSA‐based tests that are commonly ordered to screen for prostate cancer in the context of existing recommendations. Overlapping the period of 2011 to 2017 of this study, annual PSA testing rates have been reported for some U.S. men younger than age 65 using electronic records (2007‐2014),^10^, ^11^, ^12^, ^13^ claims data (2001‐2015),^14^, ^15^, ^16^, ^17^ and self‐reported survey data (2000‐2015).^18^, ^19^, ^20^, ^21^, ^22^, ^23^, ^24^, ^25^ None of these reports, however, has encompassed the most recent years since 2016. Also, none has used most of the diagnostic criteria we have employed for the purpose of limiting PSA testing to screening for prostate cancer. Although most of these studies have shown significant decreases in PSA testing rates for most age groups between 2008 and 2013 to 2015,^10^, ^11^, ^13^, ^15^, ^17^, ^18^, ^19^, ^20^, ^21^, ^22^, ^23^, ^24^ others have shown stable testing rates during this period.^12^, ^16^ Our analysis of PSA testing trends was from 2011, the year before the USPSTF released a recommendation against the screening of all men,^2^ to 2017, the year before it finalized its current recommendations.^4^ In so doing, we excluded encounter claims for PSA tests that may have been used for purposes such as monitoring of those with a history of prostate cancer or any other prostate‐related health conditions (see Appendix). Still, approximately 93% of all encounter claims remained after such an exclusion, implying that most PSA testing we have reported are likely to be due to screening. To our knowledge, only a few other studies have used specific procedure and diagnostic codes to exclude encounter claims that are likely to be associated with PSA testing ordered for purposes other than cancer screening.^14^, ^15^, ^26^ Also, no published study has documented increased PSA testing in 2018 and beyond as our study has shown to be the case from 2017 to 2018 for all but two age groups. Unique to this study is the use of hemoglobin testing as a surrogate marker of overall laboratory testing, given that this test has not been subject to changing guidelines. Given the lack of any significant trends in hemoglobin testing, there would be greater confidence that changes in PSA testing rate can be attributed to changes in screening guidelines. Kearns, et al. used MarketScan commercial claims and encounter data from 2008 through 2014 to determine annual PSA testing rates in men age 40 to 64,^15^ demonstrating that PSA testing decreased between 2008 and 2014. However, these investigators, aside from prostate cancer, did not distinguish PSA testing for screening, diagnostic, monitoring, or prognostic purposes, as done by others.^14^, ^26^ No studies using commercial claims data have gone beyond 2015. The unique observation made in our study for recent PSA testing trends is that from 2017 to 2018, PSA testing increased for the first time.

The PSA testing rates we have obtained are comparable to those published in previous reports. We are not aware of any studies that have assessed PSA testing rates in men age 30 to 39 as reported here. Furthermore, to our knowledge, no published report has used nationally based claims or electronic data to assess PSA testing rate beyond 2015.

When comparing PSA testing rates in 2011 to those in 2017 in each of the seven age groups, testing rate consistently decreased, ranging from 1.3% for men age 55 to 59 to 16.9% for those age 40 to 44. The greatest decreases of 13.4% to 16.9% were for the three age groups between 35 and 49. Although PSA testing rate decreased significantly (*P* ≤ .001) for these age groups (APC, −3.1% to −2.4%), showing no inflection points, downward trends (APC, −0.5% to 0.0%) were not significant for men age 30 to 34, 50 to 54 and 55 to 59 (*P* = .11‐.997). For men age 60 to 64, even though PSA testing decreased from 2011 to 2013 (APC, −3.2%; *P* = .054), testing rate showed a modestly significant upward trend from 2013 to 2017 (APC, +1.2%; *P* = .045). Using the most recently released 100%‐sample database covering claims from January 1, 2012 to April 30, 2019, except for age groups 35 to 39 and 30 to 34 (APC, −2.4% and − 0.5%, respectively) PSA testing rate increased from 2.7% for men age 40 to 44 to 10.7% for men age 60 to 64. These results are consistent with other studies up to 2015 based on the use of electronic^12^ and claims^16^ data that have shown stable PSA testing rates not much impacted by the 2012 USPSTF recommendation against PSA‐based screening.^2^ These findings seem to be in agreement with those from surveys of both clinicians^27^ and men^28^ soon after the release of the USPSTF draft and final recommendation statements in 2011 and 2012, respectively, suggesting these recommendations may encounter significant barriers for being adopted, given prevalent practice and men's favorable disposition to PSA testing.

There were consistent geographic differences in annual PSA testing rates in the 4 U.S. Census regions, with the South showing the highest PSA testing rates and the West exhibiting the lowest. Various commercial carriers may support different approaches to PSA testing, which may partly contribute to the differences observed between regions. This finding is consistent with the observations by Ong and Mandl who examined PSA testing rates in 2009 to 2015 using claims data from a U.S. commercial health insurer,^17^ showing the highest PSA testing rates in the South and the lowest rates in the Midwest and West. These findings are also consistent with PSA testing rates we have observed for men age ≥ 65 through 2015 using Medicare claims data, showing that the highest PSA testing rates were in the South (2011‐2015) and the lowest rates in the West (2003‐2015).^29^

Our findings should be considered in view of several limitations of this study. The sample of U.S. men studied is restricted to those with private health insurance coverage. Also, we captured only paid claims data using non‐random MarketScan samples of the U.S. population.^8^ Therefore, some PSA tests may have actually been performed but not captured because no claims were submitted for them or, if claimed, may not have been paid, thus contributing to lower observed PSA testing rates. The reported testing rates are based on adjudicated and paid claims data, and therefore some test orders may not be captured if the insurance claims were denied. Also, claims accessed via Treatment Pathways are excluded if the enrollee does not have drug coverage. Furthermore, our results should be considered in view of the limitation that MarketScan claims data constitute a large but non‐random sample of commercially insured U.S. population, and therefore they are not expected to be fully representative of this population. Of a population of approximately 70 million men age 30 to 64 in 2019 (https://www.statista.com/statistics/241488/population-of-the-us-by-sex-and-age), only about 7% were included in the study. As such, PSA testing rates and trends among uninsured men age 30 to 64 and those receiving government insurance (Medicaid and Medicare) cannot be deduced from these data. Nevertheless, regional distribution of the enrollees in this study reflected well the regional distribution for Americans as reported by the U.S. Census Bureau (data not shown). Temporal changes for specific laboratory tests may be impacted by changes in the utilization rate of laboratory tests in general. We used hemoglobin as a surrogate marker of laboratory test utilization, demonstrating that the testing rate was stable for all age groups and U.S. Census regions (*P* = .070‐.96). These limitations call for the need to access aggregate, nationally based electronic health data for men age < 65 to determine performed PSA testing rates.

Our data show that the impact of the USPSTF recommendation against testing^2^ seems to be modest. PSA testing rate generally increased from 2017 to 2018. This may partly reflect the impact of recent changes in the USPSTF recommendations.^3^, ^4^ Ordering of a laboratory test is a preanalytic component of the total testing process. This is a vital component of laboratory quality and diagnostic excellence, and it has been promulgated by initiatives, such as Choosing Wisely.^30^ Explorations of future PSA‐based testing rates and trends are avenues for future investigations.

## CONFLICT OF INTEREST

The authors have stated explicitly that there are no conflicts of interest in connection with this article.

## AUTHOR CONTRIBUTION

All authors had full access to the data in the study and take responsibility for the integrity of the data and the accuracy of the data analysis. *Conceptualization*, S.S., K.S., L.F., D.S.; *Data curation*, S.S., K.S., *Methodology*, S.S., K.S., L.F., D.S.; *Investigation*, S.S., K.S., L.F., D.S.; *Formal Analysis*, S.S., K.S.; *Project administration*, S.S.; *Writing ‐ Original Draft*, S.S.; *Writing ‐ Review & Editing*, S.S., K.S., L.F., D.S.; *Visualization*, S.S., K.S.; *Supervision*, S.S.; *Validation*, S.S., K.S.

## ETHICAL STATEMENT

Instituitional clearance and approval have been obtained prior to submission. Requirement for patient consent is not applicable for this study.

## Data Availability

The data that support the findings of this study are available from the corresponding author upon reasonable request.

## Appendix

**TABLE A1:** Exclusion codes used^a^

Code	Source	Description
60.21	ICD‐9	Transurethral ultrasound‐guided, laser induced prostatectomy
60.29	ICD‐9	Other transurethral prostatectomy
60.3	ICD‐9	Suprapubic/Transvesical prostatectomy
60.4	ICD‐9	Retropubic prostatectomy
60.61	ICD‐9	Local excision of lesion of prostate
60.62	ICD‐9	Perineal prostatectomy; cryoablation of prostate; cryoprostatectomy and surgery of prostate
60.69	ICD‐9	Other operations on prostate and seminal vesicles
185.0	ICD‐9	Malignant neoplasm of prostate
222.2	ICD‐9	Benign neoplasm of prostate
233.4	ICD‐9	Carcinoma in situ of prostate
236.5	ICD‐9	Neoplasm of uncertain behavior of prostate
600.00	ICD‐9	Benign hypertrophy of prostate without urinary obstruction and other LUTS
600.01	ICD‐9	Benign hypertrophy of prostate with urinary obstruction and other LUTS
600.10	ICD‐9	Nodular prostate without urinary obstruction
600.11	ICD‐9	Nodular prostate with urinary obstruction
600.20	ICD‐9	Benign localized hyperplasia of prostate without urinary obstruction and other LUTS
600.21	ICD‐9	Benign localized hyperplasia of prostate with urinary obstruction and other LUTS
600.90	ICD‐9	Hyperplasia of prostate, unspecified, without urinary obstruction and other LUTS
600.91	ICD‐9	Hyperplasia of prostate, unspecified, with urinary obstruction and other LUTS
601.0	ICD‐9	Acute prostatitis
601.1	ICD‐9	Chronic prostatitis
601.2	ICD‐9	Abscess of prostate
601.3	ICD‐9	Prostatocystitis
601.4	ICD‐9	Prostatitis in diseases classified elsewhere
601.8	ICD‐9	Other specified inflammatory diseases of prostate
601.9	ICD‐9	Prostatitis, unspecified
602.0	ICD‐9	Calculus of prostate
602.1	ICD‐9	Congestion or hemorrhage of prostate
602.3	ICD‐9	Dysplasia of prostate
602.8	ICD‐9	Other specified disorders of prostate
602.9	ICD‐9	Unspecified disorder of prostate
790.93	ICD‐9	Elevated PSA
55 801	ICD‐9	Perineal subtotal prostatectomy
55 810	CPT	Perineal radical prostatectomy
55 812	CPT	Perineal radical prostatectomy with lymph node biopsies
55 815	CPT	Perineal radical prostatectomy with bilateral pelvic lymphadenectomy
55 821	CPT	Suprapubic, subtotal prostatectomy
55 831	CPT	Retropubic, subtotal prostatectomy
55 840	CPT	Retropubic radical prostatectomy with or without nerve sparing
55 842	CPT	Retropubic radical prostatectomy with or without nerve sparing with lymph node biopsies
55 845	CPT	Retropubic radical prostatectomy with(out) nerve sparing/lymph node biopsies with lymphadenectomy
55 866	CPT	Laparoscopy, retropubic radical prostatectomy including nerve sparing/robotic assistance
0V500ZZ	ICD‐10	Destruction of prostate, open approach
0V503ZZ	ICD‐10	Destruction of prostate, percutaneous approach
0V504ZZ	ICD‐10	Destruction of prostate, percutaneous endoscopic approach
0V507ZZ	ICD‐10	Destruction of prostate, via natural or artificial opening
0V508ZZ	ICD‐10	Destruction of prostate, via natural or artificial opening endoscopic
0VB00ZZ	ICD‐10	Excision of prostate, open approach
0VB03ZZ	ICD‐10	Excision of prostate, percutaneous approach
0VB04ZZ	ICD‐10	Excision of prostate, percutaneous endoscopic approach
0VB07ZZ	ICD‐10	Excision of prostate, via natural or artificial opening
0VB08ZZ	ICD‐10	Excision of prostate, via natural or artificial opening endoscopic
0VT00ZZ	ICD‐10	Resection of prostate, open approach
0VT04ZZ	ICD‐10	Resection of prostate, percutaneous endoscopic approach
0VT07ZZ	ICD‐10	Resection of prostate, via natural or artificial opening
0VT08ZZ	ICD‐10	Resection of prostate, via natural or artificial opening endoscopic
A5422	ICD‐10	Gonococcal prostatitis
A5902	ICD‐10	Trichomonal prostatitis
C61	ICD‐10	Malignant neoplasm of prostate
D0.75	ICD‐10	Carcinoma in situ of prostate
D29.1	ICD‐10	Benign neoplasm of prostate
D40.0	ICD‐10	Neoplasm of uncertain behavior of prostate
N40.0	ICD‐10	Enlarged prostate without lower urinary tract symptoms
N40.1	ICD‐10	Enlarged prostate with lower urinary tract symptoms
N40.2	ICD‐10	Nodular prostate without lower urinary tract symptoms
N40.3	ICD‐10	Nodular prostate with lower urinary tract symptoms
N41.0	ICD‐10	Acute prostatitis
N41.1	ICD‐10	Chronic prostatitis
N41.2	ICD‐10	Abscess of prostate
N41.3	ICD‐10	Prostatocystitis
N41.4	ICD‐10	Granulomatous prostatitis
N41.8	ICD‐10	Other inflammatory diseases of prostate
N41.9	ICD‐10	Inflammatory disease of prostate, unspecified
N42.0	ICD‐10	Calculus of prostate
N42.1	ICD‐10	Congestion and hemorrhage of prostate
N42.30	ICD‐10	Unspecified dysplasia of prostate
N42.31	ICD‐10	Prostatic intraepithelial neoplasia
N42.32	ICD‐10	Atypical small acinar proliferation of prostate
N42.39	ICD‐10	Other dysplasia of prostate
N42.89	ICD‐10	Other specified disorder of prostate
N42.9	ICD‐10	Disorder of prostate, unspecified
N51	ICD‐10	Disorders of male genital organs in diseases classified elsewhere
R972	ICD‐10	Elevated PSA
R9720	ICD‐10	Elevated PSA
R9721	ICD‐10	Rising PSA following treatment for malignant neoplasm of prostate
V10.46	ICD‐10	Personal history of malignant neoplasm of prostate
Z85.46	ICD‐10	Personal history of malignant neoplasm of prostate

^a^ LUTS, lower urinary tract symptom; CPT, Current Procedural Terminology; ICD, International Classification of Disease.
